# Influenza vaccination in older people: a geriatrician’s perspective

**DOI:** 10.1007/s40520-025-03086-5

**Published:** 2025-06-28

**Authors:** Nicola Veronese, Ligia Juliana Dominguez, Antonina Ganci, Gerlando Speziale, Pasquale Mansueto, Salvatore Piro, Giorgio Basile, Mario Barbagallo

**Affiliations:** 1 Geriatric Unit, Department of Medicine, University of Palermo, Palermo, Italy; 2 Saint Camillus International University of Health Sciences, Rome, Italy; 3 Department of Medicine and Surgery, University of Enna Kore, Enna, Italy; 4 Department of Clinical and Experimental Medicine, University of Catania, Catania, Italy; 5 Unit and School of Geriatrics, Department of Clinical and Experimental Medicine, University of Messina, Policlinico “G. Martino”, 98124 Messina, Italy

**Keywords:** Influenza, Vaccination, Older people

## Abstract

Influenza poses a significant threat to older adults, exacerbated by age-related immune decline and the high prevalence of chronic conditions. Despite being the most effective preventive measure, influenza vaccination rates among this population remain alarmingly low, with Italy and Europe failing to meet the World Health Organization’s target of 75% coverage for individuals aged 65 and over. This review, informed by a geriatric conference in Enna, Sicily, examines the epidemiology of influenza in older populations, the impact of frailty on vaccination, and specific considerations for patients with dementia and diabetes. High-dose (HD) and adjuvanted inactived vaccines emerge as vital tools, offering enhanced immunogenicity and efficacy tailored to the unique needs of older adults. Economic analyses underscore the high direct and indirect costs of influenza in older populations, advocating vaccination as a cost-effective intervention. The authors call for increased geriatrician advocacy, education of healthcare professionals, and tailored public health strategies to improve vaccine uptake, especially among frail individuals and those with dementia or diabetes. By addressing these gaps, influenza vaccination can significantly mitigate the clinical, economic, and social burdens of the disease in older adults. The review highlights barriers to vaccination, including healthcare workers’ hesitancy, misinformation, and logistical challenges in geriatric care settings. Enhanced vaccination strategies, particularly the use of HD vaccines, are shown to reduce hospitalizations, mortality, and healthcare costs. Furthermore, the integration of vaccination into life-course immunization policies is essential for minimizing disease burden and promoting healthy aging.

## Introduction

Influenza poses a significant health risk to older adults, who are particularly vulnerable to severe complications, hospitalizations, and mortality due to age-related immune decline and high prevalence of comorbidities [1]. Vaccination remains the most effective preventive measure, yet influenza vaccination coverage among older adults is alarmingly low in both Italy and Europe. In Italy, during the 2022–2023 season, only 58.1% of individuals aged 65 and over received an influenza vaccine, falling short of the World Health Organization’s 75% target for this population [2]. Similarly, across Europe, vaccination rates for seniors vary widely, often well below recommended levels, with an average coverage of around 44% in some regions [3]. 

Despite the high-quality evidence supporting the effectiveness and safety of influenza vaccination in older people, the rate of coverage for influenza vaccination is dramatically low in older people and the role of geriatricians to improve this issue is still largely unexplored. For this reason, at the beginning of the seasonal flu vaccination in Sicily, the three post-graduate schools of geriatric medicine in this island met in Enna, Sicily (27 September 2024). This narrative review explores influenza vaccination from a geriatrician’s perspective, addressing the challenges of improving coverage rates and discussing the advantages of high-dose (HD) and adjuvanted vaccines that better meet the specific immunological needs of older adults, summarizing the main findings of the meeting.

## Methods

This narrative review is based on discussions from a geriatric conference held on September 27, 2024, in Enna, Sicily. While this review is not systematic, literature was selected using a targeted approach to identify high-quality peer-reviewed studies by each expert and guidelines relevant to influenza vaccination in older adults. A PubMed search was conducted using keywords such as “influenza vaccination,” “older adults,” “frailty,” “dementia,” and “high-dose influenza vaccine.” Preference was given to recent publications (2010–2024), large cohort studies, meta-analyses, randomized controlled trials, and expert guidelines. Key articles’ reference lists were also screened. Inclusion criteria were: (1) relevance to geriatric populations, (2) English-language publications, and (3) studies examining outcomes such as vaccine efficacy, safety, or economic impact. Excluded were case reports, non-peer-reviewed sources, and studies focused exclusively on paediatric or general adult populations.

The review was shaped by an expert meeting involving geriatricians from the three postgraduate schools of geriatric medicine in Sicily (Palermo, Messina and Catania) in collaboration with experts of Enna. Although the meeting did not formally follow a Delphi or structured consensus method, it was guided by an expert-led discussion approach. The panelists presented recent data, clinical experiences, and regional vaccination efforts, followed by thematic discussions moderated by senior geriatricians. While influenza exacerbates a broad range of chronic conditions common in older adults—including cardiovascular disease and chronic kidney disease—the expert panel chose to focus primarily on dementia and diabetes for several reasons. Firstly, these two conditions present unique challenges to influenza vaccination efforts: individuals with dementia face notable barriers such as cognitive impairment and caregiver-related decision-making, while older adults with diabetes experience altered immune responses and an increased risk of severe influenza-related complications. Secondly, during the expert meeting, local data and experiences in geriatric outpatient and inpatient settings revealed significant gaps in vaccination coverage among these two populations, highlighting them as priority targets for intervention. Although cardiovascular disease and CKD are acknowledged as important comorbidities, the panel prioritized conditions where tailored strategies might yield the greatest immediate impact.

The synthesis of evidence and expert inputs was conducted descriptively. Expert opinions and local data from vaccination initiatives were used to support narrative themes, identify barriers, and formulate potential strategies to enhance vaccination uptake. Consensus was achieved through open discussion, with informal agreement reached on key messages and recommendations.

The meeting included eight experts specializing in geriatrics, internal medicine, endocrinology, and infectious diseases. All participants were academic clinicians with experience in geriatric care and vaccine advocacy. Participants were drawn from the Universities of Palermo, Enna Kore, Catania, and Messina. The group also included researchers involved in recent vaccination initiatives targeting frail older inpatients and outpatients.

### Epidemiology of influenza in older people

Well-established data highlight the severe burden of influenza among older adults, including elevated hospitalization and mortality rates [4–10]. 

Influenza surveillance is integral to managing and preventing outbreaks. Italy’s RespiVirNet surveillance system (previously called Influnet), supported by public health institutions and sentinel physicians, provides critical data on the timing, spread, and intensity of influenza activity nationwide [11]. The system monitors influenza-like illness (ILI) cases weekly, enabling public health officials to anticipate epidemic peaks and guide vaccination efforts [11]. During the 2023–2024 flu season, peak influenza incidence occurred in the 52nd week, with older adults (65+) experiencing an incidence rate of 11 cases per 1,000, substantially lower than younger demographics, due to higher vaccination uptake among seniors [11]. 

One of the most important points is the role of healthcare workers (HCWs) in influenza transmission, noting that infection rates among unvaccinated HCWs range between 7.1% and 26% annually, with 42% of HCWs failing to recognize respiratory symptoms related to influenza [12]. Studies show that increasing influenza vaccination coverage among HCWs correlates with reduced laboratory-confirmed influenza cases and decreased nosocomial infections. For instance, raising HCW vaccination rates from 4 to 67% has been associated with a reduction in laboratory-confirmed influenza cases from 42 to 9% and a near-elimination of hospital-acquired infections [12]. This evidence underscores the need for stringent vaccination policies for HCWs to protect vulnerable patients, particularly the elderly, within healthcare settings.

Furthermore, given the overlapping respiratory nature of both diseases during winter and autumn, i.e., influenza and COVID-19, co-administration is considered safe and effective, providing robust protection during peak viral seasons. Studies confirm that immune responses to both influenza and SARS-CoV-2 spike protein are unaffected by simultaneous vaccination, supporting the development of combined influenza-COVID-19 vaccines to enhance convenience and uptake among older populations [13]. 

In conclusion, the role of epidemiological surveillance in pre-empting influenza-related public health challenges is essential. Surveillance, in fact, allows for real-time monitoring of strain circulation and epidemic severity, informing vaccine formulations and immunization timing. These data-driven insights are fundamental in refining public health strategies and minimizing influenza’s impact on older people.

### Frailty and vaccinations during older age

Frailty is a common condition among older people, affecting more than 10% of the population aging more than 65 years [14]. This condition is usually defined as a reduced reserve against acute events, such as influenza [15]. 

Given the physiological changes associated with aging, including immunosenescence, older individuals are especially vulnerable to respiratory infections, which remain a leading cause of death globally. According to WHO data, lower respiratory tract infections are the fourth leading cause of death worldwide, with significant impacts in older populations due to weakened immunity and increased comorbidities [16]. The European Union has reported approximately 27,600 annual deaths from respiratory conditions related to influenza among the elderly in 28 countries [17]. 

Immunosenescence, or the age-related decline in immune function, is identified as a critical factor reducing the efficacy of vaccines among older people [18, 19]. Immunosenescence affects both the innate and adaptive immune systems, resulting in a weaker response to infections and a decreased response to vaccines [18]. Research indicates that, with advancing age, there is a reduction in the number and efficacy of T-helper cells and B cells, essential components of the immune response [20]. Consequently, this diminished immunological capacity results in lower antibody production following standard-dose influenza vaccination, highlighting the need for enhanced vaccine formulations for older populations [21]. Recent studies show that older adults respond less effectively to standard-dose influenza vaccines, with antibody responses 2 to 4 times lower compared to younger adults for antigens like H1, H3, and B [22, 23]. 

Another important point is the connection between influenza and non-respiratory complications in older people, which contributes significantly to the disease’s overall burden. Influenza is linked to increased risks of major adverse cardiovascular events, including myocardial infarction and stroke [24], and exacerbates conditions, such as diabetes [25]. Moreover, among older adults hospitalized with influenza, studies have documented increased risks of fractures and falls, often leading to long-term functional impairments and reduced independence. For example, a Swedish cohort study showed that elderly patients hospitalized for influenza had a 28% higher risk of fractures and fall-related injuries compared to their peers [26]. 

For all these reasons, vaccination against influenza should be considered as part of a life-course immunization strategy, being crucial for promoting healthy aging and minimizing the impacts of infectious diseases in older people [27]. Vaccination should be incorporated as a core component of preventive health services in geriatric care to combat the high burden of vaccine-preventable diseases in this population, particularly to avoid the transition from frailty to disability and from disability to mortality [28]. In this regard, the authors of this conference have proposed an active vaccination campaign among older inpatients and outpatients. In hospital, for example, the proportion of patients vaccinated against influenza during hospitalisation was 62.5%, an increase of 16% in influenza vaccination uptake among frail people in comparison with the previous influenza season [29]; among older frail outpatients, mainly affected by cognitive and metabolic disorders, we observed an increase in influenza vaccination of 19.5% increase compared to the previous season [30]. Therefore, moving toward a prevention-focused healthcare model for older adults, with immunization as a key pillar, can significantly mitigate age-associated disease risks and improve quality of life and all geriatricians and physicians involved in the care of older people could be actively involved.

### Influenza vaccinations in older people: the example of dementia and diabetes

Although influenza exacerbates a broad range of chronic conditions common in older adults—including cardiovascular disease and chronic kidney disease—the expert panel chose to focus primarily on dementia and diabetes for several reasons. Firstly, these two conditions present unique challenges to influenza vaccination efforts: individuals with dementia face notable barriers such as cognitive impairment and caregiver-related decision-making, while older adults with diabetes experience altered immune responses and an increased risk of severe influenza-related complications. Secondly, during the expert meeting, local data and experiences in geriatric outpatient and inpatient settings revealed significant gaps in vaccination coverage among these two populations, highlighting them as priority targets for intervention. Although cardiovascular disease and CKD are acknowledged as important comorbidities, the panel prioritized conditions where tailored strategies might yield the greatest immediate impact.

It is estimated that every three seconds one person in the world will develop dementia [31, 32]. Recently, among the possible risk factors for dementia, increasing attention was given to infections, such as influenza, pneumococcal pneumonia, herpes zoster, and respiratory syncytial virus (RSV) that pose, per se, serious health risks to individuals with dementia [33, 34]. In this regard, it was reported by several studies that influenza vaccination is able to significantly decrease the risk of dementia in observational cohort studies [35–39]. This protective effect is present also for other vaccinations [40]. 

Having in mind this literature, it is important to note that some barriers still exist for vaccinations in older people affected by dementia [41]. Three main barriers are of importance, i.e., 1) intra-personal, such as cognitive decline and stigma, (2) inter-personal, such as caregiver distress, and (3) extra-personal, such as social isolation or financial restraints with each influence containing several subsequent sub-themes [41]. In fact, it is reported that dementia is an important factor in reducing the influenza vaccination coverage rate among older people living in the community [42]. 

Comprehensive vaccination protocols for older people living with dementia are necessary, emphasizing that immunization serves as a key preventive strategy to counteract the dual challenge of cognitive impairment and infection susceptibility. By prioritizing vaccinations tailored to the specific needs of dementia patients, healthcare providers can reduce the incidence of severe infections, mitigate declines in cognitive and physical health, and ultimately improve the quality of life for this vulnerable population.

Similarly, diabetes is of critical importance for the influenza vaccination among older patients. Diabetes, in fact, significantly increases susceptibility to infections due to immune system alterations, which heighten the risk of complications and mortality from diseases like COVID-19 and influenza [43, 44]. 

Influenza is a prominent example of the complications arising in diabetic patients. As above mentioned, the WHO estimates that influenza affects 3 to 5 million people globally each year, with excess mortality rates among those aged 65 and older reaching 223 per 100,000 inhabitants [45]. In diabetic patients, influenza often leads to severe complications, including a 7.4% increased risk of pneumonia, a 5.7% increased risk of sepsis, and a 2.1% higher likelihood of ischemic cardiac events compared to non-diabetic controls [46]. These figures underline the need for influenza vaccination as a preventive measure in managing infection risks among older diabetic patients.

It is important to detail the pathophysiological mechanisms underlying the increased infection risk in diabetes. Hyperglycemia can suppress immune responses, facilitating viral and bacterial proliferation, and increasing susceptibility to respiratory and urinary tract infections [47]. For example, hyperglycemia in the airways can lead to an alteration in bronchial secretions and cytokine production, further weakening immune defenses. This immunosuppressive effect of high glucose levels is particularly evident in respiratory infections, where diabetic patients have a significantly higher risk of complications, such as exacerbated COPD and cardiovascular mortality [48]. 

Therefore, it is important to emphasize the beneficial impact of influenza vaccination on reducing hospitalizations and mortality in older diabetics. A large randomized trial, including a consistent part of diabetic patients, showed that vaccination reduced the risk of all-cause mortality by 17%, hospitalizations by 50%, and re-hospitalizations by 17%, for example [49]. Another important study, conducted over nine consecutive influenza seasons, demonstrated a clear reduction in adverse outcomes for diabetic patients, supporting the effectiveness of annual vaccination in reducing the healthcare burden for this vulnerable population [50]. These findings were also confirmed by a large systematic review and meta-analysis using the GRADE (i.e. a systematic and transparent method for assessing the certainty of evidence), suggesting that influenza vaccination is able to decrease mortality rate in older people with diabetes [51] as well as influenza vaccination is able to significantly decrease the rate of cardiovascular conditions, also in people affected by diabetes [52]. 

WHO and various scientific societies, including the American Diabetes Association (ADA), recommend vaccinations for influenza, pneumococcus, COVID-19, and herpes zoster for older and diabetic individuals [53, 54]. Moreover, these guidelines indicate that, among all vaccines available against influenza, the best choice is the HD quadrivalent inactivated influenza vaccine due to a better stimulation of the immune system compared to the other possibilities [53]. 

Despite these clear evidences, the rate of older people affected by diabetes that will get the vaccination remains low: it is estimated that only one third of these people are keen to receive the vaccination [55]. This also probably due to the low percentage of physicians getting the vaccination and managing diabetes every day [55]. 

Therefore, it is imperative for the role healthcare professionals play in advocating for vaccinations in older diabetic patients. Vaccination can lower hospitalization rates, reduce healthcare costs, and improve quality of life by preventing infection-related complications. Proactive promotion of vaccination within this population is essential for improving public health outcomes and reducing the healthcare burden.

### The economic burden of influenza in older people

The economic burden of influenza is another important aspect to take into consideration. As mentioned before, people older than 65 years, experience higher hospitalization and mortality rates due to influenza, underscoring the necessity of targeted preventive measures, particularly vaccination [17, 56]. Influenza-associated morbidity and mortality are notably higher among older people due to comorbid conditions like cardiovascular diseases, diabetes, and chronic respiratory diseases. These comorbidities exacerbate the effects of influenza and contribute to the higher mortality rates observed globally [57]. In addition to clinical risks, influenza imposes a substantial hospital burden. Studies from Spain, Germany, and China have consistently shown that influenza hospitalizations and intensive care unit (ICU) admissions are significantly more frequent and severe in older populations [58]. These data suggest an important economic burden attributable to influenza, even if these works practically analysed only direct costs.

A seminal systematic review conducted to assess the quality-adjusted life years (QALYs) and patient-reported outcomes (PROs) affected by influenza in older adults noted significant reductions in health-related quality of life (HRQoL) following severe influenza episodes [59]. Briefly, this systematic review reports that higher direct costs were reported for people at increased risk of influenza-related complications compared to those at low risk and that older age was associated with an increased occurrence and longer duration of certain influenza symptoms finally giving an important increase in both direct and indirect costs [59]. 

At the same time, vaccination is of importance in reducing the clinical and economic burden of influenza in older people. Several studies demonstrate that older individuals who receive influenza vaccines, particularly HD or adjuvanted vaccinations, have lower rates of hospitalizations and ICU admissions than those who are unvaccinated [59]. Furthermore, studies show that influenza vaccination reduces both direct and indirect costs, highlighting its importance as a cost-effective intervention for this population [59]. 

However, it is important to underline that data gaps persist, particularly regarding the long-term humanistic impacts of influenza on quality of life and emotional health among older people [59]. Most current studies focus on the immediate clinical burden rather than the lasting effects on HRQoL and functional outcomes, such as the capacity to perform daily activities post-infection. Addressing these research gaps is essential for developing comprehensive health strategies and enhancing health technology assessments focused on elderly care [59]. 

In conclusion, influenza is associated with a disproportionately high clinical, economic, and humanistic burdens among older people. Influenza vaccination stands as a key preventive measure to alleviate these burdens by reducing the risk of severe disease, hospitalizations, and related economic costs. Expanding research into the humanistic effects of influenza on older adults is necessary to inform policies and ensure a holistic approach to healthcare that prioritizes the older population.

### Appropriate vaccine use: high-dose influenza vaccine for older people

Influenza imposes a multidimensional burden on older populations, including heightened risks of respiratory complications, cardiovascular events, strokes, and diabetes-related issues [60]. These complications, which can be life-threatening, emphasize the necessity of targeted vaccination strategies, particularly HD influenza vaccines, which are designed to address the reduced immune responsiveness in older adults. The document from the Italian Ministry of Health “*Prevenzione e controllo dell’influenza: raccomandazioni per la stagione 2024–2025*” identifies the high-dose inactivated influenza vaccine (HD-IIV) and the MF59^®^-adjuvanted inactivated influenza vaccine (adj-IIV) as the two recommended options for the population aged ≥ 65 years. HD-IIV is a quadrivalent split-virus vaccine containing two type A strains (H1N1 and H3N2) and two type B strains. It contains 60 µg hemagglutinin per strain (a fourfold increased amount of hemagglutinin per strain compared with standard-dose inactivated influenza vaccines [SD-IIV]) to ensure a greater immune response and therefore increased efficacy. Adj-IIV is a quadrivalent vaccine containing MF59 ^®^ as adjuvant, an oil-in-water emulsion of squalene oil. The adjuvant is designed to promote an adequate immune response while using a reduced amount of antigen [61]. 

A recent Italian study, conducted in the Liguria region, evaluated the effectiveness of various vaccines (standard dose vaccine, adjuvanted vaccine, and high-dose vaccine) in reducing hospitalizations for laboratory-confirmed influenza in older adults [62]. The results of this study showed that among the three vaccine types, HD-IIV4 showed statistically significant VE in most analyses (VE adjusted for age, sex, week, ≥ 1 co-morbidity and previous season vaccination = 58; IC95% 1, 82). The effect size for aIIV4 was similar (VE adjusted for age, sex, week, ≥ 1 co-morbidity and previous season vaccination = 56; IC95% −22, 84) but the 95% CI was wider and include null value. The adjusted VE of eIIV4 was generally < 25% (VE adjusted for age, sex, week, ≥ 1 co-morbidity and previous season vaccination = 2; IC95% −117, 56).

HD influenza vaccine, containing increased antigen levels (60 micrograms per strain), have been shown to significantly improve immunogenicity and reduce influenza-related outcomes compared to standard-dose vaccines [63]. A randomized Phase III trial demonstrated that HD vaccines induce a stronger immune response in adults aged 60 and above compared to standard-dose options, aligning with the heightened immunogenicity needs in older populations [64]. This response translates to a lower incidence of laboratory-confirmed influenza cases. HD vaccine is currently the only influenza vaccine globally licensed for use in the elderly population to have demonstrated greater efficacy in preventing laboratory-confirmed influenza, compared with standard-dose inactivated influenza vaccines, in an RCT [65]. Moreover, HD vaccines consistently outperform standard-dose vaccines in reducing hospitalization and mortality across multiple influenza seasons [64]. 

Clinical evidence supports the efficacy of HD vaccines in reducing hospitalizations for respiratory and cardiovascular complications in elderly individuals. A systematic review and meta-analysis covering 12 influenza seasons and more than 45 million older subjects showed that HD vaccines reduced hospitalizations 11.2% for influenza, by 14.7% for respiratory causes, and 12.8% for cardiovascular complications compared to standard-dose vaccines [66]. Notably, this reduction in hospitalizations extends across all influenza strains and seasons, even when antigenic mismatch occurs, highlighting the robustness of HD vaccines for high-risk populations [66]. 

Moreover, recent high-quality literature supports the use of HD vaccine in older people. The DANFLU-1 trial, a pragmatic randomized study conducted in Denmark, further corroborates the benefits of HD vaccines [67]. This real-world trial demonstrated that HD influenza vaccines reduced influenza- and pneumonia-related hospitalizations by 64.4% and all-cause mortality by 48.9% compared to standard-dose vaccines among adults aged 65 and older [67]. Moreover, a post-hoc analysis of the DANFLU-1 trial indicated a 70% reduction in recurrent hospitalizations for pneumonia or influenza, confirming the vaccine’s protective effect against multiple admissions due to respiratory infections in this age group [68]. These findings affirm the public health impact of HD vaccination in preventing serious outcomes and recurrent hospitalizations among older populations.

HD vaccines also offer a critical advantage in managing influenza among elderly patients with diabetes. In a post-hoc analysis of the DANFLU-1 trial, HD vaccines were associated with a significant reduction in all-cause hospitalizations for diabetic patients, highlighting the vaccine’s role in mitigating infection-related complications in this subgroup [69]. Given that older diabetics are at an elevated risk for both respiratory and cardiovascular complications from influenza, HD vaccines represent an essential preventive tool in reducing hospital visits and associated healthcare costs for this vulnerable group [69]. 

The safety and tolerability of HD vaccines are well-established, with local reactions, such as mild to moderate pain at the injection site, resolving within a few days post-vaccination [70]. This favorable safety profile, coupled with HD vaccines’ effectiveness in preventing severe influenza-related outcomes, positions HD influenza vaccination as a critical component of preventive healthcare for frail older people [65]. 

It is important to remember that current evidence suggests that HD influenza vaccines in older and high-risk groups, given their superior effectiveness in reducing hospitalizations, mortality, and serious complications compared to standard-dose vaccines. The evidence supports HD vaccination as an essential public health measure for mitigating influenza’s impact on frail older individuals and improving overall healthcare outcomes during influenza seasons.

## Discussion

During the meeting, an insightful discussion emerged around the pivotal role of correct information in healthcare professionals, particularly regarding vaccines. Experts emphasized that educating healthcare providers, especially those who work directly with vulnerable populations, is essential for strengthening public trust and ensuring effective vaccination strategies. The conversation was made around three critical aspects, i.e., empowering doctors with accurate information, curbing misinformation on social media, and enhancing vaccine awareness in nursing home environments. First, the panellists highlighted the importance of equipping doctors with up-to-date, evidence-based information on vaccines. With rapidly evolving vaccine research, including new boosters and the introduction of mRNA technology, doctors must stay informed. Misinformation not only affects patient outcomes but also undermines the credibility of healthcare providers [71]. Well-informed doctors are more capable of confidently addressing patients’ questions and concerns, providing clear guidance, and building trust. Several experts suggested regular workshops and digital resources as ways to keep healthcare professionals updated on new developments and best practices.

The second point of emphasis was on the role of social media in the spread of vaccine misinformation. As speakers noted, inaccurate information circulating on social platforms can quickly reach a wide audience, often amplifying fear and vaccine hesitancy [72]. Strategies were proposed to counter this, including targeted campaigns on popular platforms, collaboration with trusted healthcare influencers, and active fact-checking to debunk myths as they arise. By promoting reliable sources, the healthcare community can help steer public discourse toward accuracy.

Finally, the discussion turned to vaccine awareness among healthcare workers in nursing homes, where residents are particularly vulnerable. Panellists noted that these professionals are often stretched thin and may not always receive regular updates on vaccine guidelines or training in vaccine counselling. By prioritizing awareness and information flow in nursing homes, healthcare systems can better protect older nursing home residents, who are often at higher risk for vaccine-preventable diseases. Proposals included specialized training sessions and direct access to online portals with the latest vaccine-related resources.

## Conclusions

Despite the strong rationale for influenza vaccination in older populations, our review identifies several areas where further evidence is critically needed. Firstly, while vaccine efficacy in preventing hospitalization and mortality is well-documented, there is limited research on the long-term impact of influenza and its prevention on older adults’ functional status, emotional well-being, and health-related quality of life (HRQoL) post-infection. Secondly, more high-quality, prospective studies are needed to assess vaccine effectiveness among specific frail subpopulations, including those with dementia, advanced diabetes, or multiple comorbidities. In our opinion, tailored trial designs could improve precision in understanding benefits within these vulnerable cohorts. Finally, an important topic emerged during the meeting is that real-world studies on the behavioral, systemic, and informational barriers to vaccination uptake—especially in nursing homes and among caregivers—are sparse. Implementation science could help identify and evaluate scalable strategies to improve vaccine delivery and acceptance in geriatric settings.

## Data Availability

No datasets were generated or analysed during the current study.
